# Study on the Surface Layer Properties and Fatigue Life of a Workpiece Machined by Centrifugal Shot Peening and Burnishing

**DOI:** 10.3390/ma15196677

**Published:** 2022-09-26

**Authors:** Agnieszka Skoczylas, Kazimierz Zaleski

**Affiliations:** Department of Production Engineering, Faculty of Mechanical Engineering, Lublin University of Technology, 36 Nadbystrzycka, 20-618 Lublin, Poland

**Keywords:** slide burnishing, ball burnishing, centrifugal shot peening, surface roughness, surface topography, microhardness, residual stress, fatigue life

## Abstract

This paper presents the results of research on the impact of finishing method on surface topography, surface roughness (parameters *Ra*, *Rt*, *Rpk*, *Rk*, *Rvk*), surface layer microhardness, residual stresses and fatigue life. Ring samples made of C45 steel were used to conduct the experiments. The following finishing machining methods were selected: slide burnishing, ball burnishing, centrifugal shot peening, centrifugal shot peening + slide burnishing and centrifugal shot peening + ball burnishing. In the first stage, the use of combined shot peening + burnishing enables microhardness to be increased on the surface layer, the values of residual stresses to be increased and the creation of characteristic machining traces on the surface, the so-called “dimples” (effect of centrifugal shot peening). On the other hand, burnishing (slide burnishing or ball burnishing) is aimed at smoothing the surface and providing favorable stereometric properties to the surface layer. It was noted that, after finishing, the surface roughness parameters decreased from 59% to 83% in relation to the reference surface. The exception is the centrifugal shot peening technology. The use of burnishing (slide or ball burnishing) after centrifugal shot peening reduces the surface roughness parameters by a maximum of 82% compared to the value after centrifugal shot peening. The highest increase in microhardness was obtained after centrifugal shot peening + slide burnishing (*ΔHV* = 105 HV 0.05), while the highest thickness *g_h_* (*g_h_* = 120 μm) was obtained after centrifugal shot peening + ball burnishing. The combination of centrifugal shot peening and ball burnishing results in the highest absolute value of compressive residual stresses *σ_max_* = 602 MPa and depth *g_σ_* = 0.41 mm). Application of an additional operation after centrifugal shot peening increases fatigue life from 27% to 49%. ANOVA analysis of variance confirms the significance of the processing effect of centrifugal shot peening combined with slide burnishing (CSP + SB) and centrifugal shot peening + ball burnishing (CSP + BB) on the analyzed dependent surface.

## 1. Introduction

Finishing is used to ensure required dimensional and shape accuracy and appropriate properties of the surface layer of machined objects. A popular finishing method for components is shot peening. Examples of shot-peened components are gears [1], fan blades [2] and compressor discs of aero-engine [3]. The impact of a shot peening medium (usually in the form of balls) on the treated surface causes hardening of the surface layer of a workpiece and formation of compressive residual stresses [4,5].

Changes in the surface layer condition caused by shot peening have an impact on the performance properties of treated workpieces. A particularly favorable effect can be observed with respect to fatigue life [6,7]. A significant increase in fatigue limit was obtained by shot peening DIN 34CrNiMo6 alloy steel [8]. The use of shot peening also increased both the static and fatigue strength of a self-piercing riveting joint [9]. A study [10] investigated the effect of vibration-rotational shot peening on the fatigue life of C45 steel specimens. This study also analyzed the fatigue life of samples that underwentwear after shot peening. Shot peening also affects the wear and corrosion resistance of workpieces [11,12]. Residual stress relaxation caused by shot peening has a beneficial effect on fretting fatigue [13].

The impact of shot peening balls causes dimples to be formed on the treated surface. Depending on the dimple formation pattern, a distinction is made between random and regular shot peening [14,15]. In random shot peening, dimples are formed irregularly and at different distances from each other. A frequently used variation of this treatment is shot peening with a stream of balls ejected from a pneumatic device [16,17]. A variation in regular shot peening is centrifugal shot peening. This process consists of hitting the treated surface with balls, which, under the action of a centrifugal force, move freely along the axis of holes radially arranged in the rotating head [18,19]. When the technological parameters of centrifugal shot peening are constant, the dimples created by the impact of the balls are distributed over the treated surface at constant distances from each other. Application of centrifugal shot peening of the surface after laser cutting improves the properties of the surface layer of the workpiece and removes oxides formed during laser cutting [18]. Relatively soft materials, such as aluminum alloy [20], as well as hard materials [21] can be subjected to this treatment. The low values of normal forces occurring in the centrifugal shot peening (of an order of a few N) make it possible to process parts with low stiffness by this method [20]. The impact of the machining medium in the form of metal or ceramic fibers on the machined surface can be observed in the brushing process. During this treatment, the condition of the surface layer is changed and the edges of the workpiece are shaped [22,23].

In burnishing, a smooth tool is pressed with a constant force against the workpiece, and the tool moves in a tangential direction to the burnished surface. The working part of the burnishing tool can roll over the treated surface or move along the surface in a sliding manner. Depending on the shape of the working part of the burnishing tool (roller or ball), a distinction is made between roller burnishing [24,25] and ball burnishing [26,27]. On the other hand, the process in which the tool works in sliding contact with the treated surface is known as slide burnishing [28]. The working part of the tool used in slide burnishing is usually made of diamond and has the shape of a sphere with a small radius [29,30]. As with shot peening, burnishing leads to formation of compressive residual stresses in the surface layer of workpieces and increases the microhardness of this layer [31,32]. In another study [33], it was noticed that, after roller burnishing C45 steel, the plastically deformed surface layer can be divided into two characteristic zones. A surface layer is characterized by a large deformation of the grains of the metallographic structure and a large increase in hardness. The subsurface area is characterized by less visible deformations of the microstructure, with a small increase in hardness in relation to the undeformed material. An advantage of burnishing, especially slide burnishing, is that it allows obtaining a low surface roughness. Using the appropriate technological parameters of slide burnishing for normalized carbon steel, the surface roughness parameter Ra was below 0.2 µm [34]. On the other hand, the authors of another study [30] obtained the surface roughness of 0.03 ÷ 0.18 µm after cryogenic diamond burnishing of 17-4PH stainless steel. Due to changes in the surface layer properties of the surface layer, burnishing affects fatigue life [35]. A study [36] investigated the influence of machining, mirror-polishing, shot peening and low-plasticity burnishing on the high-cycle fatigue strength of DIN 34CrNiMo6 alloy steel. It was found that, compared to machined samples, the fatigue limit of shot-peened samples was 39% higher, while that of low-plasticity burnished samples increased by 52%. The burnishing-induced increase in surface layer hardness, reduced surface roughness and improved material share of the surface have a positive effect on wear resistance and lead to reduction in the coefficient of friction [37,38]. The burnishing operation, which forms a regular microrelief on the surface of the hydraulic cylinder, can also be used as a finishing treatment for such components. This method of finishing allows formation of a lubricating film on the mating surface of the tribopair “rod-hydrocylinder liner” [39,40]. According to the authors of Ref. [41], a significant increase in the resistance to fretting fatigue of modular orthopedic implants made of Ti6Al4V titanium alloy occurred as a result of low plasticity burnishing. The tribological aspects of the ball and milling burnishing process were analyzed in Ref. [42]. A significant reduction in surface roughness due to ball burnishing contributes to improved corrosion resistance [43]. After ball burnishing C45 steel, it is possible to reduce the surface roughness from 3.51 μm to 0.61 μm and to increase the hardness from 202 HB to 236 HB using this process [43]. Both experimental and numerical methods are used to study the burnishing process [44,45].

To improve functional properties of treated elements, various finishing methods are combined. A study described in Ref. [46] showed that ASTM A-36 steel treated with slide burnishing and powder pack boriding was more resistant to corrosion than only boride-treated steel. The authors of another study [47] found that use of a magnetic assistant in the ball burnishing process of C45 steel decreased the frictional resistance and caused formation of oil pockets, which improved the tribological properties of the treated surface. Moreover, the hardness of C45 steel after magnetic-assisted ball burnishing (MABB) machining can be improved by 20% [47]. Improvement in the tribological properties of AISI 1045 steel was achieved by using vibration-assisted ball burnishing. This treatment reduced friction by 39.4% and wear by 15.9% compared to ball burnishing without vibration [48]. Applying the braking moment to the roller during burnishing is an effective method for increasing the depth of the plastically deformed layer. By using this technology, it is possible to increase the thickness of the deformed surface layer of C45 steel by 30% in relation to the classic burnishing method. The beneficial feature of this method is the strengthening of shaft material by cold work, which improves its fatigue life [49]. The use of tangential and vertical ultrasonic vibration in the burnishing process of aluminum alloy reduced the surface roughness and increased the microhardness of the surface layer [50]. A combination of ball burnishing and hydroxyapatite coating is a good way to increase the corrosion resistance and immune response of AZ31B magnesium alloy [51]. A significant reduction in the surface roughness of the titanium alloy dedicated to production of biomedical implants was achieved as a result of the ball-burnishing-assisted electric discharge cladding process [52]. A combination of shot peening and ultrasonic sprayed graphene oxide coating reduced the coefficient of friction and wear of high-strength S960 steel [53]. Increased corrosion resistance and improved mechanical properties of AZ31 magnesium alloy were obtained by applying plasma spray WCCrCNi coating and shot peening [54].

Another paper [55] presented the results of a study on the effects of combining various processing methods (namely conventional shot peening, severe shot peening and re-shot peening) and a study on the surface roughness, hardness and fatigue properties of AISI 1050 steel. The use of shot peening for nodular cast iron samples that were previously laser-quenched improved the distribution of residual stresses, hardness and wear resistance [56]. In Ref. [57], it was found that the use of shot peening and subsequent vibration finishing led to increased fatigue properties, both at room temperature and elevated temperature, compared to those that were obtained only after shot peening.

Changes in the surface layer of a workpiece, both in shot peening and burnishing processes, depend on the technological parameters of these processes. The values of these parameters may be limited due to the shape of the workpiece. Such limitations apply to the contact force in the burnishing treatment for low stiffness elements. As stated above, very low force values occur in centrifugal shot peening, while a very low surface roughness, with relatively low contact force, can be obtained after slide burnishing. The aim of this study was to investigate the effect of combined centrifugal shot peening and slide burnishing on the properties of the surface layer and fatigue life of a steel workpiece. Tests were also carried out for a combination of centrifugal shot peening and ball burnishing.

## 2. Materials and Methods

Ring specimens made of C45 steel (marked according to EN ISO 683-1:2018) were used in the tests. This steel grade is used in the production of medium-loaded components of machines and devices, such as spindles, shafts, axles, unhardened gears, discs, levers and wheel hubs. The C45 steel grade is very often heat-treated in order to improve ductility and tensile strength. This steel grade is not subjected to nitriding and is difficult to weld due to crack formation in the heat-affected zone. Table 1 shows the chemical composition and selected properties of the tested material. The specimens used in the experiment had the shape of thin-walled rings with the following dimensions: external diameter *D_e_* = 56 mm, inner diameter *D_i_* = 50 mm, width *b* = 10 mm.

The pre-treatment of the C45 steel samples involved grinding with an aloxite grinding wheel. After that, the external surfaces of the ring samples were subjected to finishing. The following treatment methods were applied:

(a) slide burnishing (SB),
(b) ball burnishing (BB),
(c) centrifugal shot peening (CSP),
(d) centrifugal shot peening + slide burnishing (CSP + SB),
(e) centrifugal shot peening + ball burnishing (CSP + BB).

**Figure 1:**
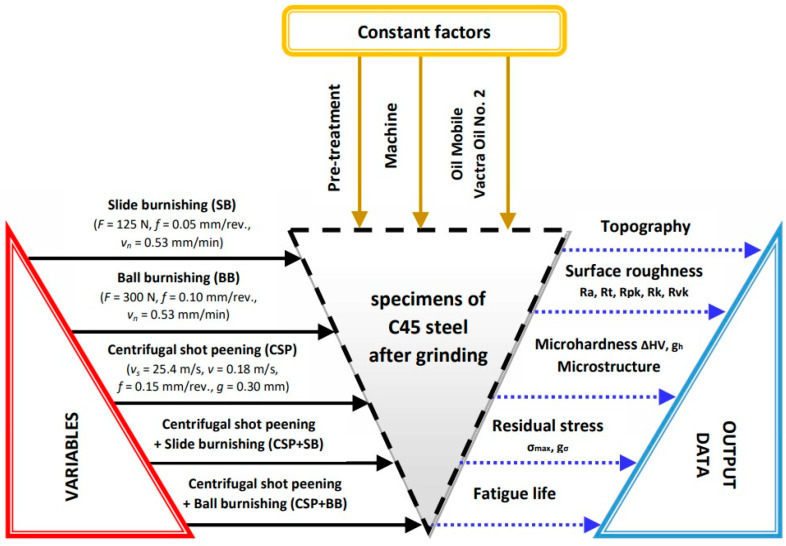
Schematic diagram of the experiment carried out.

A combination of shot peening and burnishing (e.g., centrifugal shot peening + ball burnishing) ensures favorable physical properties of the surface layer in the first step (increased microhardness, higher absolute value of compressive residual stresses and their deposition depth), and, in the next stage, it improves stereometric properties of the surface layer (low surface roughness, high material share, favorable topography). Figure 1 shows a schematic diagram of the completed experiment. Moreover, designations of the applied finishing methods for the tested C45 steel samples on the schematic diagram were presented.

Ball burnishing, slide burnishing and centrifugal shot peening were performed on a universal C11/MB machine made in Bulgaria. To enable treatment of thin-walled rings, the specimens were mounted on a mandrel that performed a rotary motion with a speed *n*, while the tools moved with a feed motion *f*.

**Figure 2:**
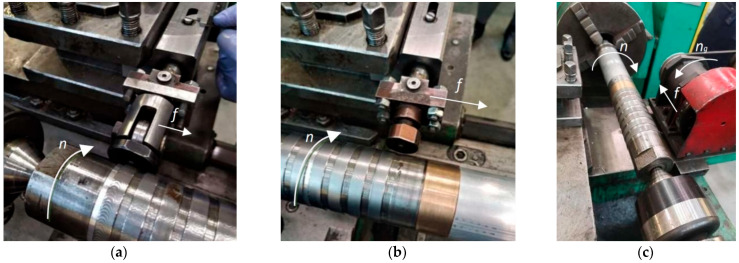
View of the stands and tools used in the experiment: (**a**) ball burnishing, (**b**) slide burnishing, (**c**) centrifugal shot peening (*f*—feed, *n*—rotational speed of the sample, *n_g_*—rotational speed of the head).

Ball burnishing (BB) (Figure 2a) was performed using a burnishing tool, which consisted of an assembly exerting the burnishing force and a burnishing element, which was a Si_3_N_4_ silicon nitride ball with a diameter of *d* = 8 mm. The following burnishing conditions were applied:

- burnishing force *F* = 300 N,
- ball burnishing feed *f* = 0.1 mm/rev.,
- burnishing speed *v_n_* = 0.53 m/s.

A slide burnishing tool (Figure 2b) was also made from a burnishing-force-exerting mechanism and a slide burnishing tip. The slide burnishing tip with a spherical shape with a radius of R = 3 mm was made of synthetic diamond. The slide burnishing process was performed with the following parameters:

- slide burnishing force *F* = 125 N,
- slide burnishing feed *f* = 0.05 mm/rev.,
- slide burnishing speed *v_n_* = 0.53 m/s.

For centrifugal shot peening (CSP) (Figure 2c), a centrifugal head with an external diameter of *D_g_* = 170 mm was used, with 27 balls having a diameter of *d_k_* = 7 mm on the circumference.

CSP was performed using the following technological parameters:

- tangential speed of centrifugal shot peening head *v_g_* = 25.4 m/s
- tangential speed workpiece *v* = 0.18 m/s
- centrifugal shot peening feed *f* = 0.15 mm/rev.
- infeed *g* = 0.30 mm.

The slide burnishing, ball burnishing and centrifugal shot peening processes were performed with the use of synthetic oil Mobile Vactra Oil No. 2

**Figure 3:**
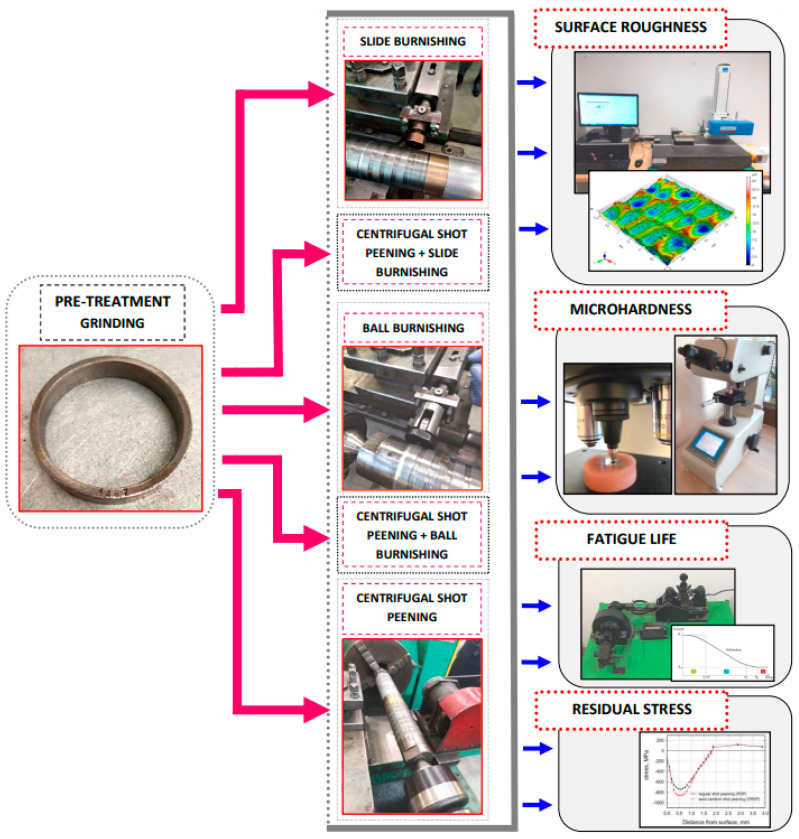
Research methods.

Following the treatment of the external surfaces of the C45 samples, selected properties of the surface layer were examined, as shown in Figure 3.

Surface roughness and surface topography measurements were conducted using the Hommel-Etamic T800 RC120-400 device. Measurements were carried out along the external the cylindrical surface of the samples (perpendicular to the treated traces) in compliance with EN ISO 25178-2:2022 (3D parameters) and EN ISO 21920-1:2022 (2D parameters). The scanned surface area was 3 × 3 mm. The analyzed 2D parameters of the surface roughness profile were: *Ra*, *Rt*, *Rpk*, *Rk*, *Rvk.*

**Figure 4:**
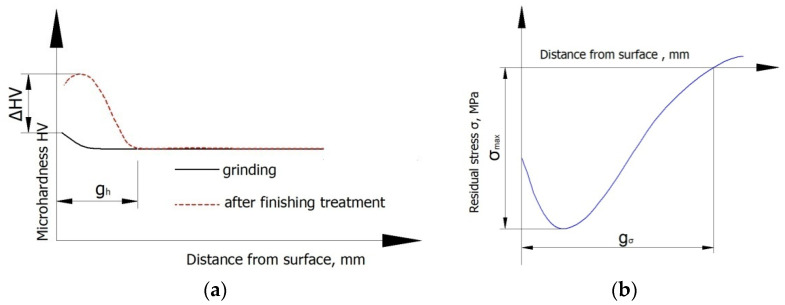
Methodology for determining: (**a**) microhardness increase *ΔHV* and hardened layer thickness *g_h_*; (**b**) the maximum absolute value of the compressive residual stresses *σ_max_* and depth of incidence of the residual compressive stress *g_σ_*.

Microhardness measurements were made in accordance with EN ISO 21920-1: 2022 on angled microsections after the standard treatment using the Vickers method. The use of angled microsections makes it possible to measure microhardness at smaller depths. At each distance from surface, the measurement was performed 10 times. Then, the extreme values (maximum and minimum value) were rejected, and the mean value and standard deviation were calculated. The LM 700at microhardness tester (Leco, St. Joseph, MI, USA) was used to that end. The indentator weight was set to 50 g (HV 0.05). Obtained microhardness results made it possible to determine a microhardness increase, *ΔHV*, and a hardened layer thickness, *g_h_* (Figure 4a). Microstructure was analyzed using the scanning electron microscope (SEM, Phenom-World, Waltham, MA, USA).

Residual stresses were measured by the destructive method. During the removal of layers with residual stresses, the deformation of the test specimen was measured. Obtained deformations made it possible to calculate residual stresses. The “removal” of the material layers was conducted by chemical etching in a 4% solution of nitric acid. Obtained data made it possible to determine the absolute maximum compressive residual stresses *σ_max_* and the depth of incidence of the compressive stresses *g_σ_* (Figure 4b).

**Figure 5:**
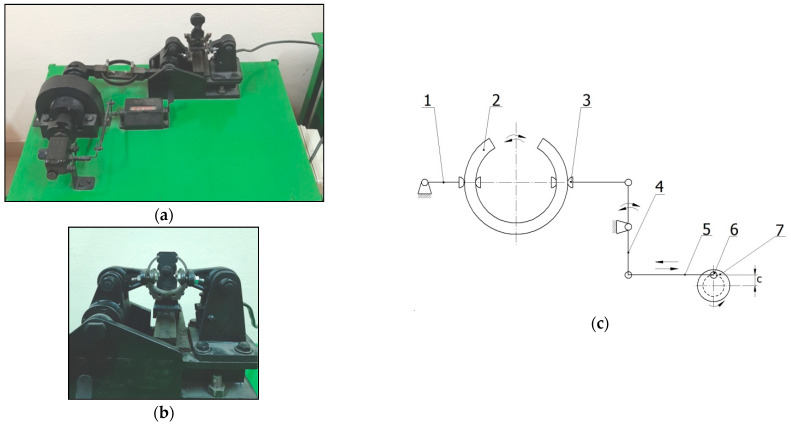
View of the test stand for fatigue life (**a**); mounted sample (**b**) and scheme of the test stand for fatigue life (**c**), where 1—fixed holder, 2—sample, 3—movable holder, 4—lever, 5—connecting rod, 6—mandrel, 7—disk.

Fatigue life tests were performed on a test stand, which is shown in Figure 5a. During tests, the following parameter was used: amplitude on the crank A = 3.45 mm, which affects the cyclic deformation of the sample. The device has a built-in counter, which allowed to record the number of sample deformations until it breaks. In the experiment, the samples were deformed cyclically so that their diameter decreased. Before the experiments, the samples were cut twice along the generating line in order to remove its fragment (Figure 5b). The sample (2) was mounted in the fixed (1) and movable (3) holders. After that, the disc (7), in which the mandrel (6) was mounted at a distance *c* from the axis, was rotated. The connecting rod (5) mounted on the mandrel (6) was moved. The lever (4) and the movable handle (3) transferred the displacement of the connecting rod (5) to the sample (2), which caused its cyclical deformation (Figure 5c). A measure of fatigue life was the amount of specimen deformation until breakage.

An analysis of variance (ANOVA) was performed to show the significance of the effect of the applied finishing technique on the roughness parameters (*Ra*, *Rpk*), microhardness increase (*ΔHV*), hardened layer thickness (*g_h_*), maximum residual stress (*σ_max_*) and depth of residual stress (*g_σ_*). The significance of the performed finishing treatment on the fatigue life of the samples was also analyzed. ANOVA was selected for surface roughness parameters *Ra* and *Rpk* because the *Ra* parameter is the most widely used parameter in engineering practice, whereas *Rpk* allows assessment of the nature of interaction between two elements. The analysis was carried out using Statistica, version 13. First, the normality of distribution was tested using the Shapiro–Wilk test, and then the homogeneity of variance was tested using the Leven test. The assumed significance level for all tests was α = 0.05. The values of the F statistics were compared with the critical value of F_α_ for the adopted level of significance and degrees of freedom. The influence of individual independent variables (type of technique used) was checked using the post-hoc test (Tukey’s test).

## 3. Results and Discussion

### 3.1. Surface Topography

Table 2 presents the surface topography and 3D parameters of the C45 steel samples after grinding (pre-treatment—Table 2 (a) and after finishing (Table 2 (b–f)). The surface irregularities formed after grinding have a regular shape. The surface irregularities have a rectilinear, unidirectional pattern (Table 2 (a)). The intervals between successive elevations have a similar distance. The resulting surface topography should be classified as unidirectional parallel. The surface topography changes after BB, SB and CSP. As a result of BB (Table 2 (b)), the elevations of the surface irregularities are flattened. The maximum surface height *Sz* in relation to its value after grinding is reduced by more than four times. Local peaks occur on the surface after SB (Table 2 (c)). The values of the *Sa*, *Sz* and *Sp* parameters are slightly higher than after ball burnishing. This is most likely due to a high coefficient of friction and strong adhesive interaction between the C45 unalloyed steel and diamond tip material of the burnishing tool. Characteristic dimples appeared on the surface after CSP, which results from the balls hitting the workpiece (Table 2 (d)). The numerous dimples on the surface of the workpiece may constitute potential “lubrication pockets”, which may have a positive effect on the tribological properties of the workpiece. The 3D parameters of the surface roughness after CSP are higher compared to the reference surface. The use of SB or BB after CSP causes changes in the surface topography (Table 2 (e,f). The maximum height of the surface irregularities is reduced, and the tops of the profile are smoothed and rounded. There is no “complete” surface deformation, which is confirmed by the presence of numerous depressions. The *Sa* parameter in relation to the centrifugal shot-peened surface decreased by three to six times, while the *Sv* parameter decreased by three to five times.

### 3.2. Surface Roughness

**Figure 6:**
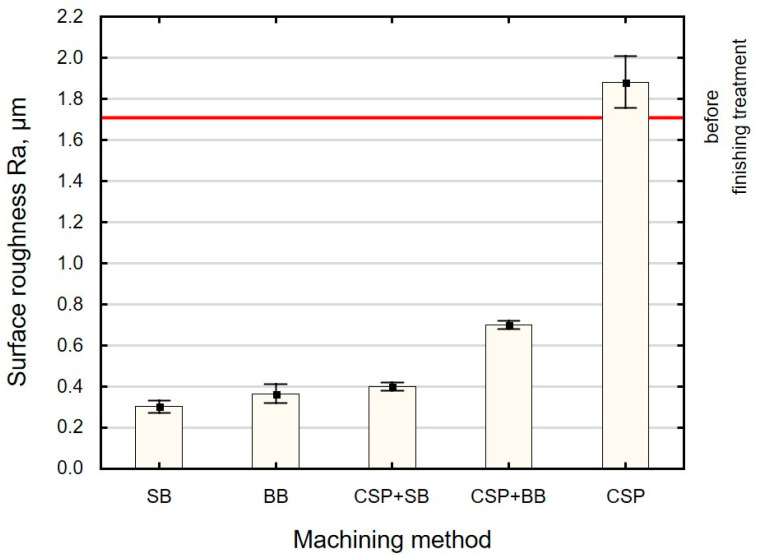
Effect of the finishing method on the surface roughness parameter *Ra* (SB—slide burnishing, BB—ball burnishing, CSP + SB—centrifugal shot peening + slide burnishing, CSP + BB—centrifugal shot peening + ball burnishing, CSP—centrifugal shot peening).

The effect of the finishing method on the roughness parameter *Ra* is shown in Figure 6. The *Ra* parameter in relation to its value after grinding (horizontal red line in Figure 6) decreased from 59% to 82% depending on the treatment method (except for CSP). CSP increased the value of the *Ra* parameter. The greatest changes in the surface roughness value were obtained as a result of SB and BB. A “constant” contact of the burnishing element with the workpiece causes “full” deformation of surface irregularities after grinding, which results in obtaining a surface of good quality. The values of the *Ra* parameter obtained after SB are identical to the results presented in Ref. [34], while, after BB, they are lower than the results described in Ref. [18]. On the other hand, application of an additional operation after CSP generates positive results. This is due to the re-deformation of the same surface irregularities. The re-deformation of the irregularities already pre-deformed by CSP causes a “more complete” change in the shape and height of the profile. The use of SB after CSP reduced the *Ra* parameter by 79% and the use of BB by 63%.

**Figure 7:**
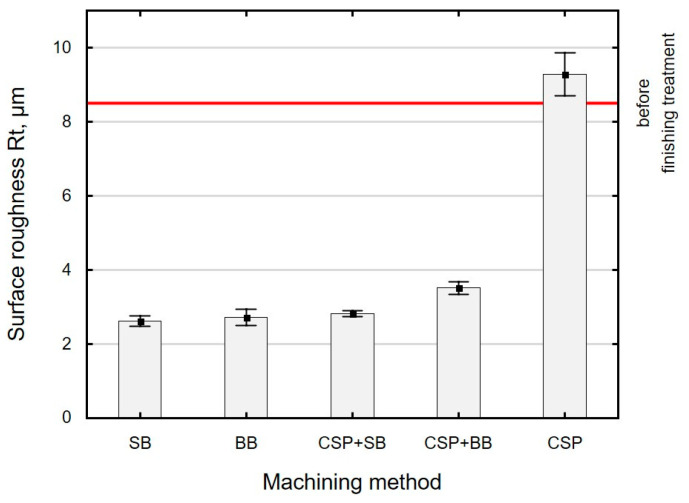
Effect of the finishing method on the surface roughness parameter *Rt* (SB—slide burnishing, BB—ball burnishing, CSP + SB—centrifugal shot peening + slide burnishing, CSP + BB—centrifugal shot peening + ball burnishing, CSP—centrifugal shot peening).

Similar changes in the function of the applied finishing treatment were obtained for the surface roughness parameter *Rt* (Figure 7). The use of SB after CSP made it possible to obtain *Rt* parameter values similar to those obtained with BB or SB alone. However, the use of two finishing methods most likely has a positive effect on the physical properties of the surface layer. The *Rt* parameter in relation to the surface after grinding decreased from 59% to 69% depending on the applied method of treatment. After CSP, the total height of the surface roughness profile increased by 9% compared to the value after grinding. This is most likely due to the nature of interaction (hitting) between the balls and the treated surface.

**Figure 8:**
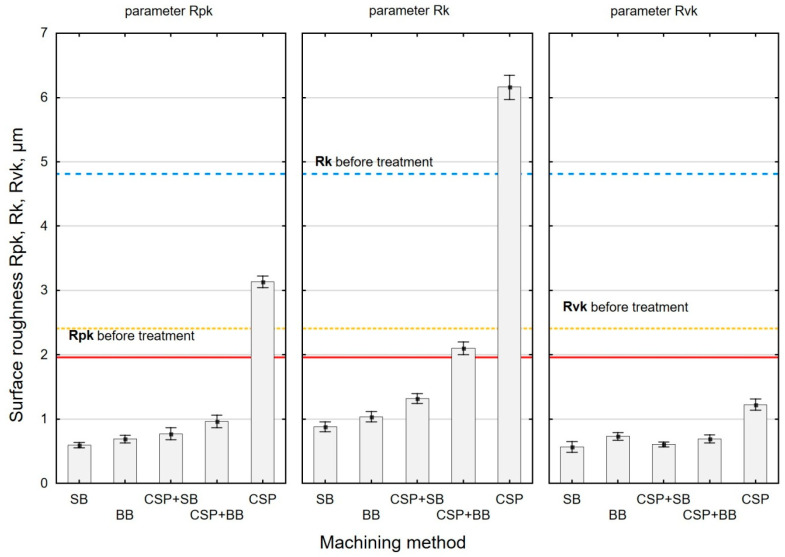
Effect of the finishing method on the surface roughness parameters *Rpk*, *Rk*, *Rvk* (SB—slide burnishing, BB—ball burnishing, CSP + SB—centrifugal shot peening + slide burnishing, CSP + BB—centrifugal shot peening + ball burnishing, CSP—centrifugal shot peening).

In terms of interaction between two surfaces, the parameters related to the Abbott–Firestone curve are of vital importance. The parameters of the bearing curve describe the nature of this interaction. Figure 8 shows the effect of the finishing treatment on the roughness parameters *Rpk*, *Rk* and *Rvk*. The parameters from the *Rk* group characterize not only roughness height but also its shape. This is very important in terms of describing the functional behavior of the surface.

For all the variants of the applied finishing treatment, except for CSP, the parameter *Rpk* (reduced peak height) has lower values than after grinding. This means that the surfaces treated by these methods will be characterized by proper interaction during lapping. After CSP, the *Rk* parameter increased by 97% in relation to the reference value, while, in other cases, the values are lower by 56–83%. The lower *Rk* values mean that, after the lapping period, a significant part of the surface will be in contact with the surface of the mating element. The reduced valley depth (parameter *Rvk*) decreased after machining compared to the value after grinding. This may mean that the surfaces treated in this way may have a lower oil retention capacity.

Interestingly, the *Rvk* parameter decreased after CSP, while the *Rpk* and *Rk* parameters increased compared to their values after grinding. This can be explained by the rounding of the “valleys” of the profile through the impact of the balls, which promotes the “flow” of the material. As a consequence, there is an increase in the *Rpk* parameter. The changes in the Abbott–Firestone parameters after CSP differ from those obtained for laser-cut specimens [18]. They are higher than the values obtained after CSP of the laser-cut specimens [18]. This is probably due to the type of pre-treatment.

The Abbott–Firestone curve parameter values show similar dependencies to the *Ra* and *Rt* roughness parameters. The lowest values of the *Rpk*, *Rk* and *Rvk* parameters were obtained after SB. The highest values of *Rpk*, *Rk* and *Rvk* were obtained after CSP. However, the use of an additional operation after CSP does not generate such favorable results, as in the case of the *Ra* and *Rz* parameters. The *Rpk* parameter decreased from 69% to 75% and the *Rk* from 66% to 73% in relation to the value after CSP. Interestingly, lower values of the *Rvk* parameter are obtained after the combined CSP + SB and CSP + BB treatment rather than after SB or BB alone. This can be explained by the fact that the profile of the grinding surface is characterized by sharp depressions, while the profile of the surface after CSP has rounded valleys, which is probably favorable for the obtained dependencies.

Table 3 shows Tukey’s test results for dependent variables (roughness parameter *Ra* and *Rpk*). The analysis was possible because the ANOVA analysis of variance confirmed that the type of finishing treatment had a significant effect on the *Ra* and *Rpk* parameters. The probability level *p* is greater than the accepted significance level (α = 0.05), and the value of the test statistic F_(4; 20)_ is greater than the accepted F_α_.

The analysis shows that the application of additional processing (SB or BB) after CSP significantly influences the obtained roughness parameters *Ra* and *Rpk*. It should also be noted that there are no statistically significant differences between the roughness parameters obtained as a result of SB and CSP + SB and BB and CSP + SB. Analysis of the significance of the influence of the applied finishing treatment and the achievement of favorable values of the surface roughness parameters after the combined CSP + BB and CSP + SB treatment confirm the validity of using this type of technique as a finishing treatment for elements made of C45 steel.

### 3.3. Microhardness

**Figure 9:**
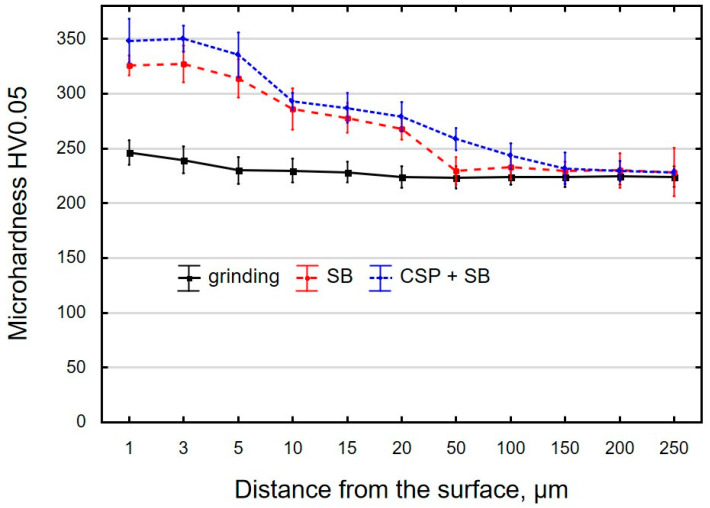
Microhardness of the surface layer in the samples after grinding (pre-treatment), slide burnishing (SB) and centrifugal shot peening and slide burnishing (CSP + SB).

As a result of finishing, the microhardness of the surface layer changes (Figure 9). During grinding, in the tool–workpiece contact zone, there is a high temperature and friction. Noticeable changes in the microhardness of the specimens after grinding occur just below the surface, at a depth of 1 μm to 3 μm. From a depth of approximately 10 μm, the microhardness of the surface layer is similar to the microhardness of the core. The use of SB causes an additional increase in the microhardness. The changes extend to a depth of approximately 50 μm. The maximum microhardness after SB is observed at a depth of approximately 3 μm from the surface. This is a zone with the most deformed crystals. The use of two techniques (CSP + SB) causes the changes in the surface layer microhardness to extend deeper (approximately 90 μm), and the increase in the microhardness is also greater.

**Figure 10:**
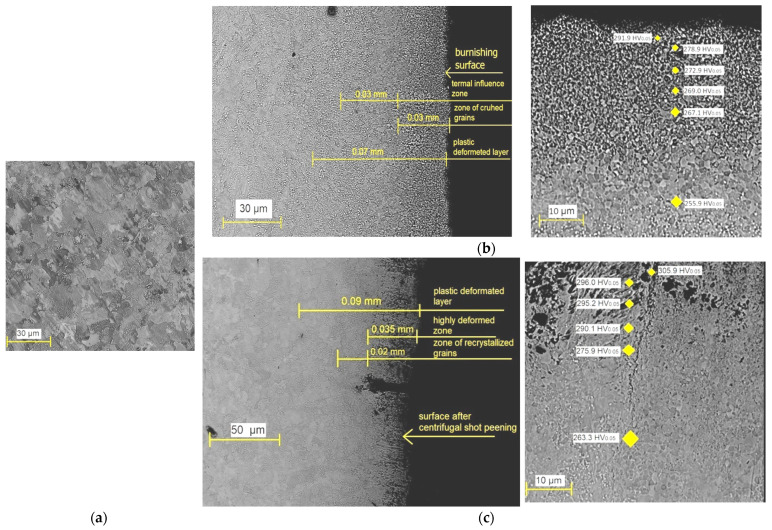
Microstructure C45 steel: (**a**) before finishing; (**b**) after BB; (**c**) after CSP.

C45 steel, before finishing treatment, has a ferritic–pearlitic structure (Figure 10a). There are visible ferrite and perlite grains. After BB (Figure 10b), there is a zone of crushed grains under the surface, created as a result of the impact of the tool. It is formed as a result of breaking the perlite grains. Visible as well is a zone of thermal influence (fine-grained structure), which is transforming into a ferritic–pearlitic structure. As a result of the hitting of the balls on the surface treated during CSP, the microstructure is changed (Figure 10c). A highly deformed zone is visible below the surface. There are discontinuities and numerous surface defects, which favors an increase in the dislocation density. Under the deformed zone, plastically deformed crystals and a zone of recrystallized grains (grinding effect) are visible. Then, the core material is visible.

**Figure 11:**
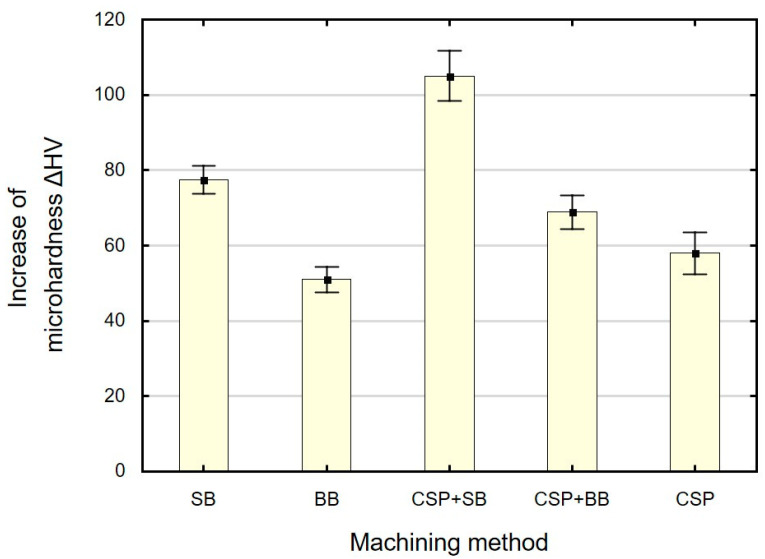
Finishing method versus increase in microhardness *ΔHV* (SB—slide burnishing, BB—ball burnishing, CSP + SB—centrifugal shot peening + slide burnishing, CSP + BB—centrifugal shot peening + ball burnishing, CSP– centrifugal shot peening).

**Figure 12:**
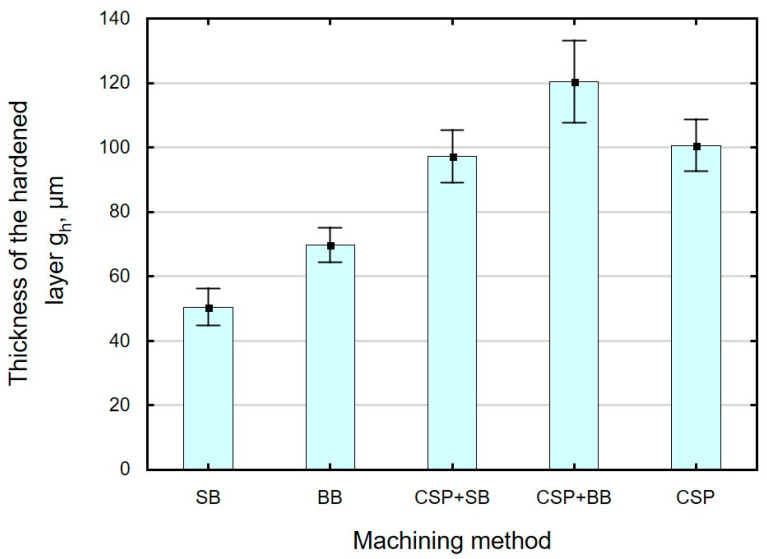
Finishing method versus hardened layer thickness *g_h_* (SB—slide burnishing, BB—ball burnishing, CSP + SB—centrifugal shot peening + slide burnishing, CSP + BB—centrifugal shot peening + ball burnishing, CSP– centrifugal shot peening).

Figure 11 shows the increase in the microhardness *ΔHV,* and Figure 12 shows the hardened layer thickness *g_h_* as a function of the applied finishing treatment. The use of CSP and SB causes the greatest increase in microhardness (*ΔHV* = approximately 105), with the thickness of the hardened layer *g_h_* being smaller than after CSP and BB. A significant increase in microhardness is obtained after SB. The obtained changes in microhardness after SB are at a similar level as those obtained after slide burnishing for X19NiCrMo4 steel (e = 32%) [58], with the thickness of the hardened layer being greater than that reported in Ref. [58]. As expected, the combination of two finishing techniques (CSP + SB and CSP + BB) causes a greater increase in the microhardness *ΔHV*, and the changes occur deeper than for the BB or SB only. The use of SB or BB after CSP leads to an increase in the crushing energy, which accumulates and thus causes greater changes in microhardness. A greater increase in *ΔHV* in relation to the microhardness value after CSP was obtained using SB. The depth of changes in *g_h_* occurring after the combined CSP + SB treatment is at a similar level as that observed after CSP. The greatest depth of changes in *g_h_* occurs as a result of the application of CSP and then BB. The obtained values of the depth of changes in the microhardness *g_h_* are greater than those described in Refs. [27,34].

An analysis of the results presented in Table 4 demonstrates that the use of BB or SB after CSP has a significant effect on the dependent variable *ΔHV*. However, there are no statistically significant differences in the hardened layer thickness *g_h_* when using BB or SP after CSP.

### 3.4. Residual Stress

**Figure 13:**
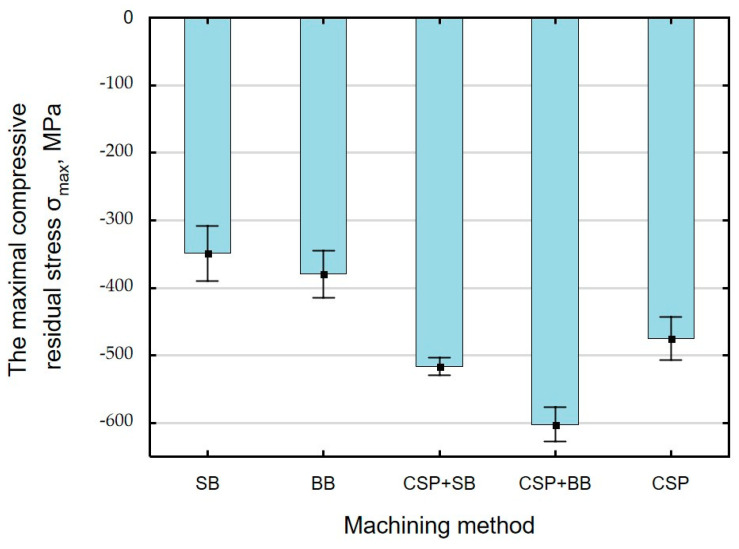
Finishing treatment versus the maximum (absolute value) compressive stresses *σ_max_* (SB—slide burnishing, BB—ball burnishing, CSP + SB—centrifugal shot peening + slide burnishing, CSP + BB—centrifugal shot peening + ball burnishing, CSP—centrifugal shot peening).

**Figure 14:**
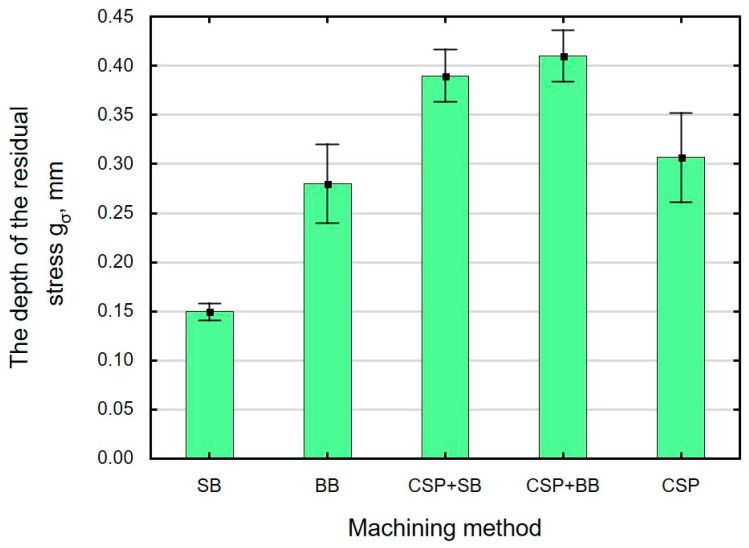
Finishing treatment versus residual compressive stresses *g_σ_* (SB—sidle burnishing, BB—ball burnishing, CSP + SB—centrifugal shot peening + sidle burnishing, CSP + BB—centrifugal shot peening + ball burnishing, CSP– centrifugal shot peening).

Compressive residual stresses are generated in the surface layer as a result of finishing. The absolute value of maximum residual stresses *σ_max_* (Figure 13) and the incidence depth *g_σ_* (Figure 14) depend on the finishing technique used.

The highest (absolute) value of compressive residual stresses was obtained after CSP + BB. For the case of a single finishing method, the highest absolute residual stresses *σ_max_* occurred after CSP, which agrees with the authors’ previous works on CSP [18]. The lowest absolute value of *σ_max_* and depth *g_σ_* were obtained after SB, which is related to the changes in microhardness depth. The depth of residual stresses and their “nature” are also related to the structural changes that take place in the surface layer during treatment [27]. The combination of CSP and SB causes the absolute stress value *σ_max_* to increase by 36% and the depth g_σ_ by 160% when compared to SB. The greatest depth *g_σ_* was obtained after CSP + BB, which is confirmed by the change in the microhardness of the surface layer (Figure 12).

A combination of two finishing techniques: CSP + SB and CSP + BB results in positive changes in the value of residual stresses *σ_max_* and the depth of their incidence *g_σ_*, which complies with the results presented in Ref. [56] for laser quenching combined with shot peening.

As expected, application of an additional operation (BB) after CSP significantly influences the absolute value of stresses *σ_max_* and the depth incidence *g_σ_* of compressive stresses (Table 5). There are no statistically significant differences in the values of stresses *σ_max_* and *g_σ_* following application of SB after CSP.

### 3.5. Fatigue Life

**Figure 15:**
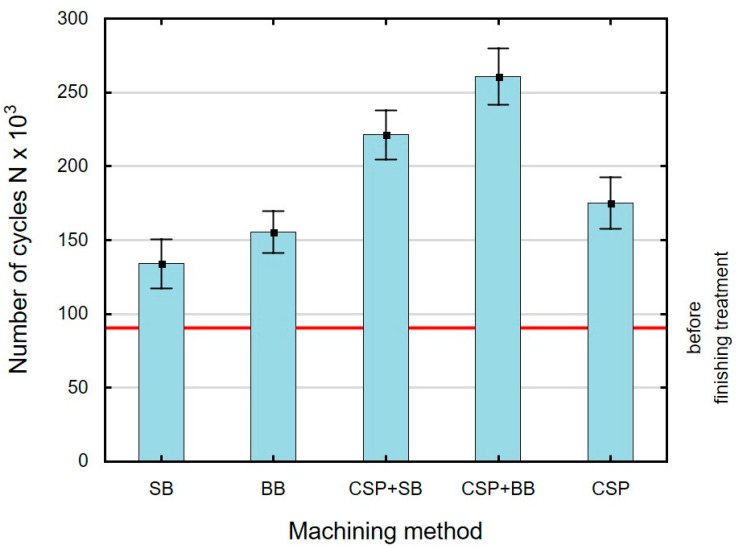
Effect of the finishing treatment on fatigue life (SB—slide burnishing, BB—ball burnishing, CSP + SB—centrifugal shot peening + slide burnishing, CSP + BB—centrifugal shot peening + ball burnishing, CSP—centrifugal shot peening).

Figure 15 shows the effect of finishing methods on the fatigue life of C45 steel ring specimens. The horizontal line indicates the durability of the specimens after grinding. The number of cycles of sample contraflexure until breakage is approximately 90,000. The fatigue life of the specimens subjected to shot peening and burnishing increased from 48% to 123% in relation to the durability values for the reference samples. The highest fatigue life was obtained after using CSP+BB. This can be explained by the greatest depth of changes in the microhardness of the surface layer *g_h_* and the greatest depth of compressive residual stresses *g_σ_*. The number of cycles N until sample breakage is related to the values of microhardness and depth *g_h_* as well as the value of *σ_max_* and depth *g_σ_*. Use of SB after CSP increases the fatigue life by about 27% in relation to CSP. On the other hand, use of BB results in an approximate 49% increase in fatigue life in relation to CSP. The increase in fatigue life after application of CSP + SB or CSP + BB is lower than that obtained after finish turning (FT) and low plasticity burnishing (LPB) (increase by 82.4%) for the samples made of Inconel 718 alloy [35] and for the samples made of DIN 34CrNiMo6 steel after shot-peening (SP) and low-plasticity burnishing (LPB) [36]. The obtained values of fatigue life prove the validity of combining CSP with SB and CSP with BB.

The ANOVA analysis of variance results for fatigue life (Table 6) show that use of an additional finishing treatment (SB or BB) after CSP has a significant effect on fatigue life.

## 4. Summary

Based on the results of the study investigating the influence of shot peening and burnishing on selected properties of the surface layer and fatigue life of C45 steel specimens, the following conclusions can be drawn:

1. Depending on the finishing method, the geometrical structure of the surface changed after grinding. After pre-treatment, the surface was deformed in a plastic way. One can observe the flattening of the peaks of surface irregularities after BB and SB and the formation of dimples on the surface after CSP.
2. The analyzed surface roughness parameters (*Ra*, *Rt*, *Rpk*, *Rk* and *Rvk*) decreased after finishing compared to their reference values (after grinding). These changes range from 59% to 83% depending on the surface roughness parameter and the technique used. The exception is CSP. After CSP, the parameters *Ra*, *Rt*, *Rk* and *Rpk* are greater than after grinding.
3. An unfavorable effect of the applied treatments is the reduction in the roughness parameter *Rvk*. This may mean that the treated surface will have lower lubricant retention.
4. The use of SB or BB after CSP reduces the roughness parameters by a maximum of 82% in relation to their values after CSP.
5. After burnishing and shot peening, the microhardness of the surface layer and the depth of the hardened layer increased. The maximum increase in the microhardness *ΔHV* was obtained after CSP + SB. However, the greatest thickness of the hardened layer was obtained after CSP + BB. Use of an additional finishing treatment after CSP causes a greater increase in microhardness and increased thickness *g_h_*.
6. Compressive residual stresses are formed in the surface layer as a result of finishing, the depth of which *g_σ_* depends on the finishing method. A combination of CSP with BB or SB causes an increase in the maximum value of residual stresses and depth *g_σ_* in relation to their values after CSP.
7. In relation to the reference samples, the fatigue life of the samples after finishing increased from 48% to 123%. Application of an additional operation after CSP increased the fatigue life from 27% to 49%.
8. The ANOVA analysis of variance and the post-hoc test results show that the finishing techniques have a significant influence on the analyzed variables (roughness parameters *Ra*, *Rpk*, values of *ΔHV*, *g_h_*, *σ_max_* and *g_σ_*). It should be noted that the significance of the impact was observed for most of the same cases. This may prove the existence of a correlation between the obtained values.
9. An analysis of the significance of impact showed that application of additional SB or BB after CSP significantly affected the dependent variables.
10. The favorable values of the analyzed properties of the surface layer and the increase in fatigue life confirm the validity of using CSP + SB and CSP + BB as finishing treatments.

## Figures and Tables

**Table 1:** Chemical composition and strength properties of C45 steel.

Chemical Composition (Average), %								
C	Mn	Si	P	S	Cr	Ni	Mo	Fe
0.48	0.78	0.36	0.011	0.01	0.09	0.02	0.002	rest
Yield point (min)						R_e_ = 430 MPa		
Tensile strength (min)						R_m_ = 740 MPa		
Hardness (min)						250 HB		

**Table 2:** Surface topography and 3D parameters of C45 steel samples after grinding and different finishing methods.

(a) after grinding		(b) after BB	
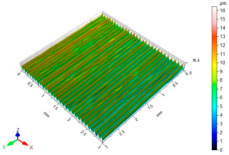	Sa = 1.96 μm Sz = 16.4 μm Sp = 9.40 μm Sv = 7.04 μm	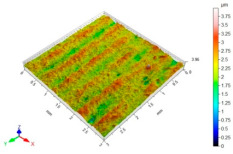	Sa = 0.22 μm Sz = 3.99 μm Sp = 1.64 μm Sv = 2.35 μm
(c) after SB		(d) after CSP	
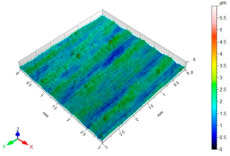	Sa = 0.33 μm Sz = 6.00 μm Sp = 4.24 μm Sv = 1.77 μm	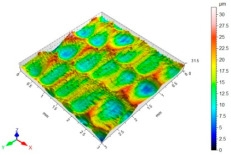	Sa = 3.01 μm Sz = 31.6 μm Sp = 15.9 μm Sv = 15.7 μm
(e) after CSP + SB		(f) after CSP + BB	
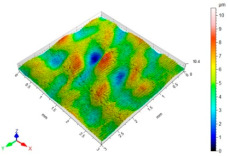	Sa = 0.91 μm Sz = 10.5 μm Sp = 5.51 μm Sv = 5.03 μm	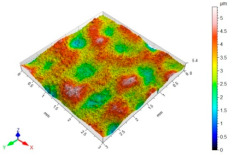	Sa = 0.54 μm Sz = 5.42 μm Sp = 2.10 μm Sv = 3.33 μm

**Table 3:** Comparative analysis of the significance of differences (post-hoc/Tukey’s test) between the mean values of the roughness parameter *Ra* and *Rpk* after treatment with various methods. The level of probability for which there are statistically significant differences is marked in red.

*Ra*					
Finishing Treatment					
	SB	BB	CSP + SB	CSP + BB	CSP
SB		0.54966	0.14948	0.00013	0.00013
BB	0.54966		0.89950	0.00013	0.00013
CSP + SB	0.14948	0.89950		0.00013	0.00013
CSP + BB	0.00013	0.00013	0.00013		0.00013
CSP	0.00013	0.00013	0.00013	0.00013	
***Rpk***					
**Finishing Treatment**					
	**SB**	**BB**	**CSP + SB**	**CSP + BB**	**CSP**
SB		0.39454	0.02185	0.00013	0.00013
BB	0.39454		0.52877	0.00035	0.00013
CSP + SB	0.02185	0.52877		0.00822	0.00013
CSP + BB	0.00013	0.00035	0.00822		0.00013
CSP	0.00013	0.00013	0.00013	0.00013	

**Table 4:** Comparative analysis of the significance of differences (post-hoc/Tukey’s test) between the average values of increased microhardness *ΔHV* and hardened layer thickness *g_h_* after treatment with different methods. The red color indicates the probability level for which there are statistically significant differences.

*ΔHV*					
Finishing Treatment					
	SB	BB	CSP + SB	CSP + BB	CSP
SB		0.00012	0.00012	0.02034	0.00012
BB	0.00012		0.00012	0.00012	0.05713
CSP + SB	0.00012	0.00012		0.00012	0.00012
CSP + BB	0.02034	0.00013	0.00012		0.00208
CSP	0.00012	0.05713	0.00012	0.00208	
***g_h_***					
**Finishing Treatment**					
	**SB**	**BB**	**CSP + SB**	**CSP + BB**	**CSP**
SB		0.10676	0.00047	0.00017	0.00032
BB	0.10673		0.01614	0.00030	0.00780
CSP + SB	0.00047	0.01614		0.04445	0.98391
CSP + BB	0.00017	0.00030	0.04445		0.09414
CSP	0.00032	0.00780	0.98691	0.09414	

**Table 5:** Comparative analysis of the significance of differences (post-hoc/Tukey’s test) between the mean values of the maximum compressive stresses *σ_max_* and the stress depth incidence *g_σ_* after treatment with different methods. The red color indicates the probability level for which there are statistically significant differences.

*σ_max_*					
Finishing Treatment					
	SB	BB	CSP + SB	CSP + BB	CSP
SB		0.73693	0.00052	0.00017	0.00363
BB	0.73693		0.00210	0.00019	0.02201
CSP + SB	0.00052	0.00210		0.03939	0.50807
CSP + BB	0.00017	0.00019	0.03939		0.00347
CSP	0.00363	0.02201	0.50807	0.00347	
***g_σ_***					
**Finishing Treatment**					
	**SB**	**BB**	**CSP + SB**	**CSP + BB**	**CSP**
SB		0.00392	0.00018	0.00017	0.00107
BB	0.00392		0.01213	0.00399	0.83985
CSP + SB	0.00018	0.01213		0.93487	0.05822
CSP + BB	0.00017	0.00399	0.93467		0.01785
CSP	0.00107	0.83985	0.05822	0.01785	

**Table 6:** Comparative analysis of the significance of differences (post-hoc/Tukey’s test) between the mean values of the fatigue life after treatment with different methods. The red color indicates the probability level for which there are statistically significant differences.

Fatigue Life					
Finishing Treatment					
	SB	BB	CSP + SB	CSP + BB	CSP
SB		0.55045	0.00074	0.000187	0.08226
BB	0.55045		0.00531	0.00027	0.63644
CSP + SB	0.00074	0.00531		0.09796	0.04410
CSP + BB	0.00018	0.00027	0.09796		0.00083
CSP	0.08226	0.63644	0.04410	0.00083	

## Data Availability

Not applicable.

## Abbreviations

BB	ball burnishing
CSP	centrifugal shot peening
CSP + BB	centrifugal shot peening + ball burnishing
CSP + SB	centrifugal shot peening + slide burnishing
*g_h_*	hardened layer thickness
*g_σ_*	the depth of incidence of the compressive stresses
*Ra*	arithmetic average of profile height deviations from the mean line
*Rk*	core roughness
*Rpk*	reduced peak height
*Rt*	total height of the profile
*Rvk*	reduced valley depth
SB	slide burnishing
*ΔHV*	microhardness increase
*σ_max_*	absolute maximum compressive residual stresses
